# Machine Learning Elucidates Electrophysiological Properties Predictive of Multi- and Single-Firing Human and Mouse Dorsal Root Ganglia Neurons

**DOI:** 10.1523/ENEURO.0248-24.2024

**Published:** 2024-10-01

**Authors:** Nesia A. Zurek, Sherwin Thiyagarajan, Reza Ehsanian, Aleyah E. Goins, Sachin Goyal, Mark Shilling, Christophe G. Lambert, Karin N. Westlund, Sascha R. A. Alles

**Affiliations:** ^1^Department of Anesthesiology and Critical Care Medicine, University of New Mexico Health Sciences Center, Albuquerque, New Mexico 87106; ^2^Department of Internal Medicine, University of New Mexico Health Sciences Center, Albuquerque, New Mexico 87131

**Keywords:** electrophysiology, human DRG, machine learning, mouse DRG, pain, sensory neuron

## Abstract

Human and mouse dorsal root ganglia (hDRG and mDRG) neurons are important tools in understanding the molecular and electrophysiological mechanisms that underlie nociception and drive pain behaviors. One of the simplest differences in firing phenotypes is that neurons are single-firing (exhibit only one action potential) or multi-firing (exhibit 2 or more action potentials). To determine if single- and multi-firing hDRG neurons exhibit differences in intrinsic properties, firing phenotypes, and AP waveform properties, and if these properties could be used to predict multi-firing, we measured 22 electrophysiological properties by whole-cell patch-clamp electrophysiology of 94 hDRG neurons from six male and four female donors. We then analyzed the data using several machine learning models to determine if these properties could be used to predict multi-firing. We used 1,000 iterations of Monte Carlo cross-validation to split the data into different train and test sets and tested the logistic regression, *k*-nearest neighbors, random forest, support vector classifier, and XGBoost machine learning models. All models tested had a >80% accuracy on average, with support vector classifier, and XGBoost performing the best. We found that several properties correlated with multi-firing hDRG neurons and together could be used to predict multi-firing neurons in hDRG including a long decay time, a low rheobase, and long first spike latency. We also found that the hDRG models were able to predict multi-firing with 90% accuracy in mDRG neurons. Understanding these properties could be beneficial in the elucidation of targets on peripheral sensory neurons related to pain.

## Significance Statement

This study will improve the understanding of the electrophysiological mechanisms of DRG neurons. Our machine learning algorithms show few species differences between mouse and human DRG neuron electrophysiology under baseline conditions. These are important findings for the study of neuronal excitability in the context of pain therapeutic development.

## Introduction

Neuronal hyperexcitability is a hallmark of chronic pain and understanding the electrophysiological mechanisms that lead to neuronal excitability is crucial for the development of pain treatments (Berta et al., 2017; Alles and Smith, 2018). Sensory neurons isolated from human and mouse dorsal ganglia (hDRG-N and mDRG-N) are important tools in the study of neuronal excitability, chronic pain, and nociception (Davidson et al., 2014; Zhang et al., 2017; Emery and Ernfors, 2018; North et al., 2019; Zheng et al., 2019; Zurek et al., 2024). While the transcriptomes of hDRG and mDRG have been compared (Ray et al., 2018), few studies have compared the electrophysiological features of these tools. Single-firing DRG-N that fire only one action potential (AP) are less excitable than multi-firing DRG-N that fire two or more APs. Using patch-clamp electrophysiology, we measured neuronal excitability of hDRG-N and mDRG-N in current-clamp mode. We then compared electrophysiological properties that determine multi- or single-firing in DRG-N using sophisticated computational approaches. The rationale for this methodology is to build upon understanding of electrophysiological properties of sensory neurons with machine learning tools.

Machine learning is becoming a popular tool in data analysis, tool refinement, and prediction in understanding molecular, electrophysiological, and physiological mechanisms in pain research (North et al., 2019; Gonzalez et al., 2021; Koos et al., 2021; Ingram et al., 2023; Nagaraja et al., 2023). In this study, we aimed to understand if there are electrophysiological differences between single- and multi-firing hDRG-N and mDRG-N and if these differences are predictive of neuronal excitability. Factors governing excitability might thereby be targets for modulation with therapies. Additionally, these improved tools can lead to automated, and therefore more efficient, ways to evaluate future electrophysiological data. The benefits of automating data analysis are better data transparency and reproducibility, while minimizing the need for obtaining additional live tissue.

In this study, we compared the electrophysiological features of multi- and single-firing hDRG-N and mDRG-N and applied several machine learning algorithms to elucidate which combinations of features were most predictive of multi-firing neurons. Because we have collected a relatively high number of hDRG-N recordings from a diverse demographic of donors (Zurek et al., 2024), we aimed to see if machine learning algorithms can predict whether an hDRG-N will be single- or multi-firing based on other intrinsic, phenotypic, and AP waveform electrophysiological features. While many machine learning studies use a single model to make predictions, we used several different models. We used Monte Carlo cross-validation (MCCV) simulations to iterate the train and test data split to obtain average model accuracies (Shan, 2022) and extract the most important electrophysiological features. Finally, we aimed to see if the machine learning models that we generated were also able to predict multi-firing mDRG-N so as to compare species differences. While we found several electrophysiology features were correlated with multi-firing cells, the machine learning models converged on just a few of those features as being the most predictive.

This study will improve understanding of the electrophysiological mechanisms of DRG neurons. Our machine learning algorithms paradoxically show few species differences between mouse and human DRG neuron electrophysiology under baseline conditions. The FDA Modernization Act 2.0 removes the requirement for use of animal models in therapeutic safety and effectiveness testing in favor of cell-based assays and computational modeling (Zushin et al., 2023). Our study is a first step into understanding the electrophysiological mechanisms governing neuronal excitability in both hDRG-N and mDRG-N using computational modeling. Understanding neuronal excitability in peripheral neurons such as the DRG is an important area of study to generate therapeutics that target peripheral mechanisms as opposed to mechanisms that target the CNS (Raja et al., 2020). We hope that this study and the computational algorithms used will apply to therapeutic discovery efforts for pain and other sensory disorders.

## Materials and Methods

### hDRG-N culture

hDRG-N culture was performed as previously described (Valtcheva et al., 2016; Zurek et al., 2024). hDRG were obtained from consenting recently deceased organ donors at University of New Mexico Hospital in coordination with New Mexico Donor Services. Study activities were approved by the Human Research Review Committee at the University of New Mexico Health Sciences Center; approval numbers #21-412 or #23-205. Cultures of hDRG-N were prepared as described previously and cultured for up to 11 d in vitro (DIV) (Zurek et al., 2024) and electrophysiological recordings took place between DIV 3 and 11. We used high-quality recordings where all 22 electrophysiological properties could be extracted from 94 untreated hDRG-N. The data from 8 of 10 donors was previously published in Zurek et al. (2024). Donor demographics are described in Table 1.

**Table 1.** Donor demographics

hDRG Donors	Sex	Age (Years)	BMI	Race	Number of recordings
1	F	22	22	White	1
2	F	51	30	White	13
3	M	23	26	Hispanic	14
4	M	67	34	Native	7
5	M	55	19	White	2
6	M	27	24	Hispanic	2
7	M	21	22	White	13
8	F	37	18	Hispanic	18
9	M	51	27	Unknown	3
10	F	23	27	Hispanic	21
94 Total recordings from 10 donors					

Human DRG tissue was obtained from ethically consented, recently deceased donors. This analysis used tissues from 4 female and 6 male donors aged 21–67 years.

### Mouse DRG cultures

All animal procedures were compliant with the NIH Guide for the Care and Use of Laboratory Animals and followed ARRIVE guidelines. Studies are approved by the Institutional Care and Use Committee of the University of New Mexico Health Sciences Center (IACUC #23-201364-HSC, 5-13-2024). Mouse DRG cultures were performed as previously described with some modifications (Kunamneni et al., 2023). Briefly, lumbar DRG were collected and put into an enzymatic solution containing sterile, magnesium/calcium-free HBSS (Invitrogen), papain (Worthington), dispase (Sigma), and collagenase (Worthington). Enzymatic digestion was carried out at 37°C, 5% CO_2_ for 40 min with trituration every 20 min. Complete DMEM-based media (2 ml DMEM supplemented with 10% fetal bovine serum, and 1% antibacterial/antimycotic with 100 units/ml of penicillin, 100 µg/ml of streptomycin, 25 µg/ml of amphotericin B) was added to the enzymatic digestion. The solution was then strained through a 100 µm cell strainer and rinsed several times with an additional 6 ml of complete media. The digested DRG cell suspension was rinsed by gentle centrifugation at 300 × *g* for 5 min and resuspended in 1 ml of complete media. A total of 125 µl of the mouse DRG cell suspension was added to each 12 mm coverslip precoated with poly-d-lysine (Neuvitro) and coated with additional 50 µg/ml laminin, allowed to attach for 30–60 min before gently flooding the wells with enough media to fill each well, 1–2 ml for a 12-well plate. Electrophysiological recordings were done 18–24 h after mDRG culture completion.

### Whole-cell patch-clamp electrophysiology

Whole-cell patch-clamp electrophysiology was performed as previously described (Zurek et al., 2024). Recordings were done at room temperature, with the recording chamber perfused with artificial cerebrospinal fluid (aCSF) containing the following (in mM): 113 NaCl, 3 KCl, 25 NaHCO_3_, 1 NaH_2_PO_4_, 2 CaCl_2_, 2 MgCl_2_, and 11 dextrose bubbled with 95% O_2_/5% CO_2_ (Zurek et al., 2024). Neurons were identified with differential interference contrast optics connected to an IR-2000 digital camera (Dage MTI) or an Olympus digital camera. Cell diameter was measured using Dage MTI camera software or ImageJ (NIH). Current-clamp recordings were performed using a MultiClamp 700B (Molecular Devices). Signals were acquired as previously described using a Digidata 1550B converter (Molecular Devices) and recorded using Clampex 11 software (Molecular Devices). Patch pipettes with electrode resistance of 3–7 MΩ were made fresh with a Zeitz puller (Werner Zeitz) from borosilicate thick glass (GC150F, Sutter Instrument). Intracellular patch pipette solution contained the following (in mM): 120 K-gluconate, 11 KCl, 1 CaCl_2_, 2 MgCl_2_, 10 HEPES, 11 EGTA, 4 Mg-ATP (Zurek et al., 2024). Cells that did not fire APs or had an RMP of greater than −35 mV were excluded from further analysis. Bridge balance was applied for all recordings. Analysis was performed in Easy Electrophysiology v.2.5.1, Clampfit 11.2 (Molecular Devices), and the Python v3.12 package pyABF (Harden, 2022). All statistical analysis was performed using GraphPad Prism v10.0.2. Error bars denote mean ± standard error of the mean (SEM) unless otherwise specified.

### Electrophysiology intrinsic properties analysis methods

Electrophysiological analysis was done as previously described and used recordings from eight human donors published in Zurek et al. (2024) and data from two additional donors not previously published. Current-clamp recordings started with 25 ms of the cell at rest, followed by 500 ms current pulse increasing in 10 pA increments from −100 pA in increments until they reached inactivation up to 4 nA, and finally 500 ms recovery between each current-clamp step. Current-clamp recordings were analyzed using Easy Electrophysiology Software and pyABF (Harden, 2022; Zurek et al., 2024). RMP was calculated from the first current-clamp recording after establishing a whole-cell configuration. The hyperpolarizing −100 pA step was used to determine input resistance (*R*_in_) and sag ratio. Rebound firing was defined as the neuron firing an AP during the 500 ms recovery in any of the hyperpolarizing current injection steps. Rheobase was defined as the first depolarizing current injection in which a neuron fired an AP; therefore, the lowest possible value for rheobase is 10 pA. The first spike latency (FSL) was determined by measuring the time of that the rheobase spike fired after the start of current injection. Cell capacitance was calculated using the whole-cell capacitance compensation circuit in MultiClamp 700B (Molecular Devices). Normalized rheobase was calculated by dividing rheobase by capacitance. Delayed firing was classified as a neuron that had an FSL > 100 ms. The presence of visible sag was identified by visual inspection. hDRG-N that fired more than one AP during any current injection step were labeled as multi-firing (65/94 for hDRG-N, 14/20 for mDRG-N) while neurons that fired only one AP were labeled single-firing (29/94 for hDRG-N, 6/20 for mDRG-N) in our classification system. Spontaneous activity was assessed in current-clamp mode with either no current injection or injecting enough current injection to hold the membrane voltage at −45 mV during a 30 s recording. If the neuron had at least one AP during the 30 s, it was recorded as having spontaneous activity. AP waveform properties were calculated using Easy Electrophysiology as previously described (Zurek et al., 2024). Only DRG-N which we were able to accurately measure all the properties were considered for further analysis. The same analysis techniques were used both hDRG-N and mDRG-N. We did not correct for liquid junction potential in our analyses.

Statistics comparing all electrophysiological properties between single- and multi-firing hDRG-N were performed in GraphPad Prism v10.0.2 using a Mann–Whitney test or Fisher's exact test.

### Pearson’s correlation matrices

Pearson’s correlation matrices for both hDRG-N and mDRG-N were generated in Python v3.12 using the Seaborn package for all electrophysiological properties described (Waskom, 2021). Jupyter Notebook was used to write and share code and PANDAS was used for data processing (McKinney, 2010; Kluyver et al., 2016).

### Machine learning methods

All machine learning methods were performed in Python v3.12 using scikit-learn (Pedregosa et al., 2011), an open-source machine learning package. Jupyter Notebook was used as a framework for writing, editing, and sharing code across unscaled, scaled, and feature-selected datasets (Kluyver et al., 2016). The open-source package, PANDAS (McKinney, 2010), was used for data processing, scaling, normalization, and analysis. Matplotlib and Seaborn data visualization libraries were used to produce figures (Hunter, 2007; Waskom, 2021). The dataset preprocessing included standardizing the dataset using the standard scaler in scikit-learn, converting categorical variables to binary variables, and feature selection. The unprocessed and preprocessed dataset was then used to tune the hyperparameters of the four models we used: logistic regression (LR), *k*-nearest neighbors (KNN), random forest (RF), support vector classifier (SVC), and eXtreme Gradient Boost (XGBoost). The tuning was accomplished using GridSearchCV with a five-fold split, a scikit-learn method that performs an exhaustive search over the given hyperparameters to determine which combination of hyperparameters generates the highest accuracy for a given dataset and model. The optimal hyperparameters were then used during the MCCV. Afterward, the dataset was inputted into MCCV for each of the hyperparameterized models. The MCCV split the original dataset into a training and testing dataset without replacement where the training dataset contains 80% of the samples and the testing dataset consists of 20% of the samples. Then, the training dataset was used to train the model, which was then applied to the testing dataset.

The predictions that the trained model creates based on the testing dataset are compared with the presence of multi-firing for those cells to produce an accuracy value. The creation of the subsets, training, and testing is repeated 1,000 times, allowing us to extract various metrics and attributes of the model across the iterations, resulting in comprehensive measures of the model that account for variations in the training and testing dataset induced by the random selection of the datasets. Utilizing the MCCV, we were able to visualize the distribution of the accuracy, precision, recall, and *F*1 values for each of the models and obtain the corresponding median, mean, standard error of the mean, and range. Additionally, by storing the coefficient or feature importance values for each of the MCCV iterations, we derived the mean weight given to each feature coefficient throughout the iterations. We also used Shapley Additive Explanations (Lundberg and Lee, 2017) to extract feature importance across all models on the 80%/20% split using MCCV. The SHAP values were plotted on beeswarm plots and then bar plots showing the absolute average SHAP values. All the code is available at https://github.com/PainLabUNM/Zurek_et_al_eNeuro_2024.

### Principal component analysis

As an alternate approach to understanding feature importance, we used the selected features of hDRG-N to perform a principal component analysis (PCA) in Python v3.12 using scikit-learn (Pedregosa et al., 2011). Jupyter Notebook was used to write and share code, and PANDAS was used for data processing and normalization (McKinney, 2010; Kluyver et al., 2016). We first normalized the data using the “normalize” function and then applied three-component analysis. We extracted the explained variance and feature importance using scikit-learn and plotted the data using matplot-lib (Hunter, 2007).

## Results

### Intrinsic properties in multi- versus single-firing hDRG-N

**Figure 1.**
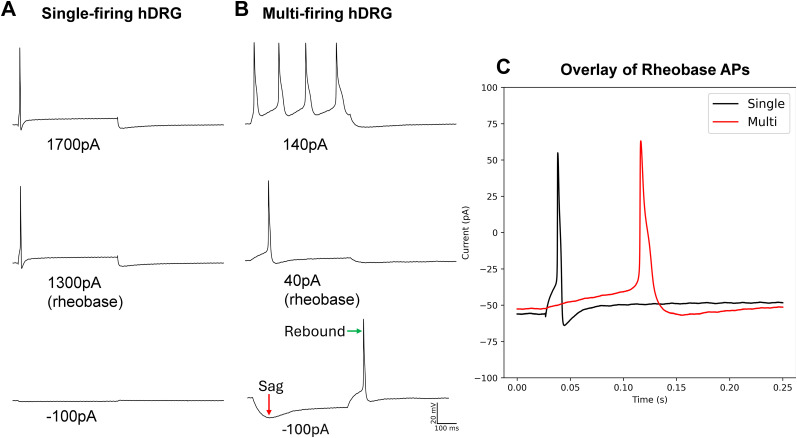
Example traces of a single-firing and multi-firing hDRG-N. ***A***, Example traces of a single-firing hDRG-N at −100 pA, rheobase, and 400 pA over rheobase. ***B***, Example traces of a multi-firing hDRG-N at −100 pA, rheobase, and at 100 pA over rheobase. Scale bar, 100 ms, 20 mV. ***C***, Zoom in of rheobase trace of cells in ***A*** and ***B*** showing the short AP duration and short FSL of the single-firing hDRG-N (black) and the long AP duration and long FSL of the multi-firing hDRG-N (red).

**Figure 2.**
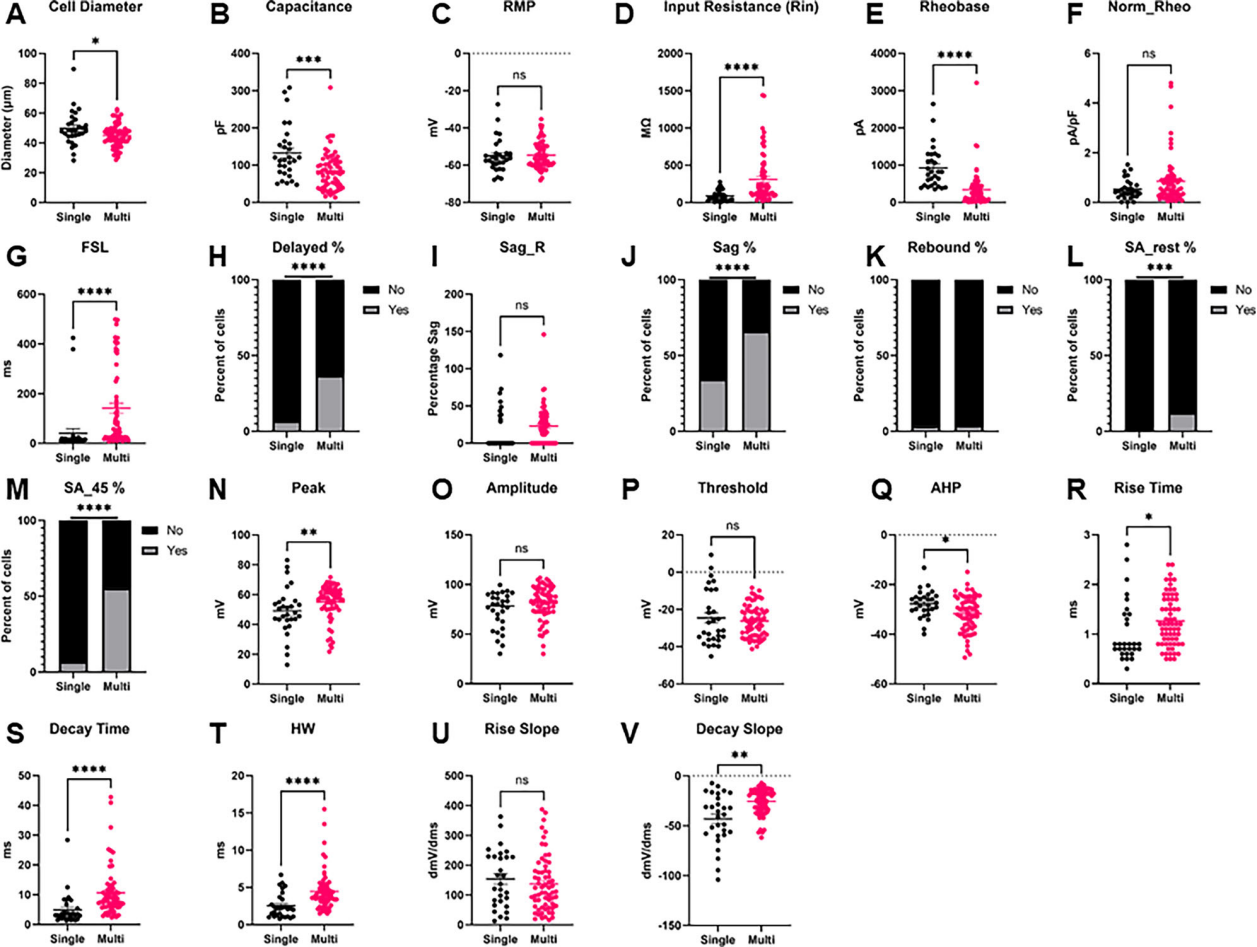
Intrinsic properties and firing phenotypes of hDRG-N neurons in single versus multi-firing cells. Intrinsic properties and firing phenotypes were compared between single and multi-firing cells. ***A***, Cell diameter, ***B***, capacitance, ***C***, resting membrane potential (RMP), ***D***, input resistance (Rin), ***E***, rheobase, ***F***, normalized rheobase, ***G***, first-spike latency (FSL), ***H***, percent delayed firing, FSL > 100 ms, ***I***, sag percentage at −100 pA current input (Sag_R), ***J***, percentage of cells with visible sag, ***K***, percentage of cells that have rebound firing. ***L***, Percentage of cells with spontaneous activity (SA) at rest, ***M***, percentage of cells with spontaneous activity when held at −45 mV. ***N***, AP peak. ***O***, AP amplitude. ***P***, AP threshold. ***Q***, AP After hyper-polarization (AHP). ***R***, AP rise time, ***S***, AP decay time, ***T***, AP half-width. ***U***, Max AP rise slope. ***V***, Max AP decay slope. *p < 0.05, **p < 0.01, ***p < 0.001, ****p < 0.0001 by Mann–Whitney for ***A–G***, ***I***, ***N–V***. By Fisher's exact test for ***H***, ***J–M***.

Using patch-clamp electrophysiology in current-clamp mode, we compared a variety of intrinsic and phenotypic properties of single- and multi-firing hDRG-N (Fig. 1, example traces ). Single-firing hDRG-N made up 30.8% (29/94) of our dataset. Cell diameter measured during clamp recording with an IR-2000 Dage MTI camera (Fig. 2*A*) showed that multi-firing hDRG-N are smaller than single-firing hDRG-N (45.03 ± 0.95 µm and 49.69 ± 2.15 µm, respectively, *p* = 0.0377). Cell capacitance was also significantly different, single-firing cells had an average capacitance 132.3 ± 13.34 pF, and multi-firing cells had an average capacitance of 84.58 ± 6.26 pF, *p* = 0.0008 (Fig. 2*B*). There was no significant difference in RMP between single and multi-firing cells (Fig. 2*C*). Single-firing cells had a significantly lower input resistance (*R*_in_), 89.23 ± 13.55 MΩ, compared with 311.2 ± 40.25 MΩ for multi-firing cells, *p* < 0.0001 (Fig. 2*D*). Rheobase for single firing cells (927.2 ± 103.8 pA) was significantly higher than rheobase for multi-firing cells (340.6 ± 58.86 pA), *p* < 0.0001 (Fig. 2*E*), though when rheobase was normalized to cell capacitance, there was no significant difference in normalized rheobase (Fig. 2*F*). FSL was sooner in single-firing cells than multi-firing cells (40.64 ± 18.63 ms and 141.50 ± 20.26 ms, respectively, *p* < 0.0001; Figs. 1*C*, 2*G*). We defined cells with delayed firing as cells with an FSL > 100 ms and multi-firing cells (37%) were much more likely to exhibit delayed firing than single-firing cells (7%) consistent with the FSL data, *p* < 0.0001 (Fig. 2*H*). There was no significant difference in sag percentage (Fig. 2*I*); however, multi-firing cells were more likely, 66%, to show visible sag than single-firing cells, 34%, *p* < 0.0001 (Figs. 1*B*, 2*J*) on visual inspection. There was no significant difference in rebound firing (Figs. 1*B*, 2*K*). We also looked at spontaneous activity of the cells both at rest and when applying enough current to hold the cell at −45 mV membrane potential. At rest, only multi-firing cells exhibited any spontaneous activity, 12%, compared with 0% for single firing cells (Fig. 2*L*). Multi-firing cells also had more spontaneous activity when held at −45 mV, 55% of multi-firing cells compared with only 7% of single-firing cells (Fig. 2*M*).

### AP waveform properties in multi- versus single-firing hDRG-N

#### AP amplitude

Single- and multi-firing hDRG-N AP waveform properties were analyzed using whole-cell patch-clamp electrophysiology in current-clamp mode of the rheobase spike (Fig. 1*C*, example traces ). There were no significant differences found in AP amplitude or AP threshold (Fig. 2*O*,*P*). However, there was a significant difference in AP peak. Single-firing hDRG-N had a smaller AP peak of 48.95 ± 2.93 mV and multi-firing hDRG-N had an AP peak of 54.95 ± 1.46 mV, *p* = 0.0068 (Fig. 2*N*). There was also a difference in AP after hyperpolarization (AHP). Single-firing cells had an AHP of −27.77 ± 1.00 mV and multi-firing cells had and AHP of −31.79 ± 0.90 mV, *p* = 0.0219 (Fig. 2*Q*).

#### AP duration

AP duration of the rheobase in hDRG-N was analyzed and multi-firing cells had a longer AP duration in all measurements, AP rise time, AP decay time, and AP half-width (HW). AP rise time for single-firing hDRG-N was 1.045 ± 0.116 ms and 1.265 ± 0.63 ms for multi-firing hDRG-N, *p* = 0.0143 (Fig. 2*R*). AP decay time was also significantly longer in multi-firing hDRG-N at 10.64 ± 1.02 ms and shorter in single-firing hDRG-N 4.934 ± 0.984 ms, *p* < 0.0001 (Fig. 2*S*). AP HW was also longer in multi-firing hDRG-N at 4.483 ± 0.322 ms and 2.545 ± 0.308 ms, *p* < 0.0001 (Fig. 2*T*). We also looked at the maximum rise and decay slope. While there was no significant difference in max rise slope (Fig. 2*S*), the max decay slope was significantly larger in multi-firing hDRG-N (−25.64 ± 1.61 dmV/dms) compared with single-firing hDRG-N (−43.14 ± 4.69 dmV/dms), *p* = 0.0011 (Fig. 2*T*).

### Correlations of electrophysiological properties in single- versus multi-firing hDRG-N

**Figure 3.**
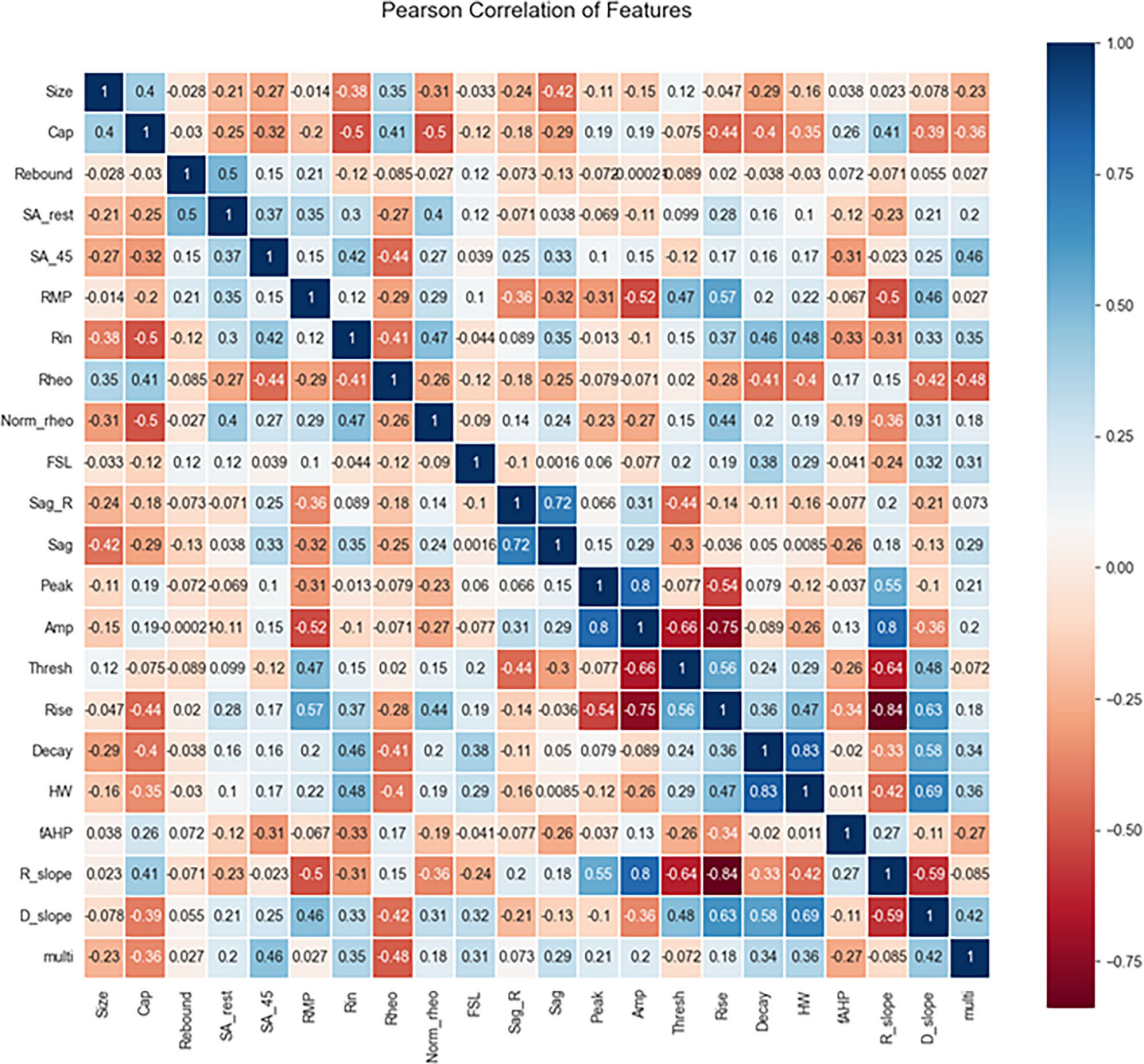
Pearson’s correlation matrix of hDRG-N features. Features compared included size, capacitance (Cap), presence of rebound firing, spontaneous activity at rest (SA_rest), spontaneous activity at −45 mV (SA_45), resting membrane potential (RMP), input resistance (Rin), rheobase (Rheo), normalized rheobase (Norm_rheo), first spike latency (FSL), sag ratio (sag_R), presence of visible sag, AP peak, AP amplitude, AP threshold, AP rise time, AP decay, AP half-width, afterhyperpolarization (fAHP), rise slope (R_slope), decay slope (D_slope), and multi-firing.

Pearson’s correlation matrix was used to determine correlations of the electrophysiological properties measured in single- and multi-firing hDRG-N (Fig. 3). Several properties were positively correlated with multi-firing cells including spontaneous activity when the cell was held at −45 mV (SA_45), *R*_in_, FSL, visible sag, long AP duration (specifically decay time and HW), and a larger decay slope. Some properties were negatively correlated with multi-firing hDRG-N including cell capacitance and rheobase. We also found some interesting correlations throughout. For example, rheobase in hDRG-N was negatively correlated with AP rise, AP decay, and AP HW, suggesting that cells that have a long AP duration tend to fire a lower current input. AP rise time was negatively correlated with AP amplitude and AP peak showing that the length of the depolarization phase of an AP affects the amplitude of the AP. Capacitance was positively correlated with cell size which has been previously reported (Zheng et al., 2019). Taken together, these data help elucidate not only the electrophysiological properties of hDRG-N that are correlated with single and multi-firing cells but also how these properties are correlated with other properties in hDRG-N.

### Comparison of model accuracies in predicting single- and multi-firing hDRG-N

**Figure 4.**
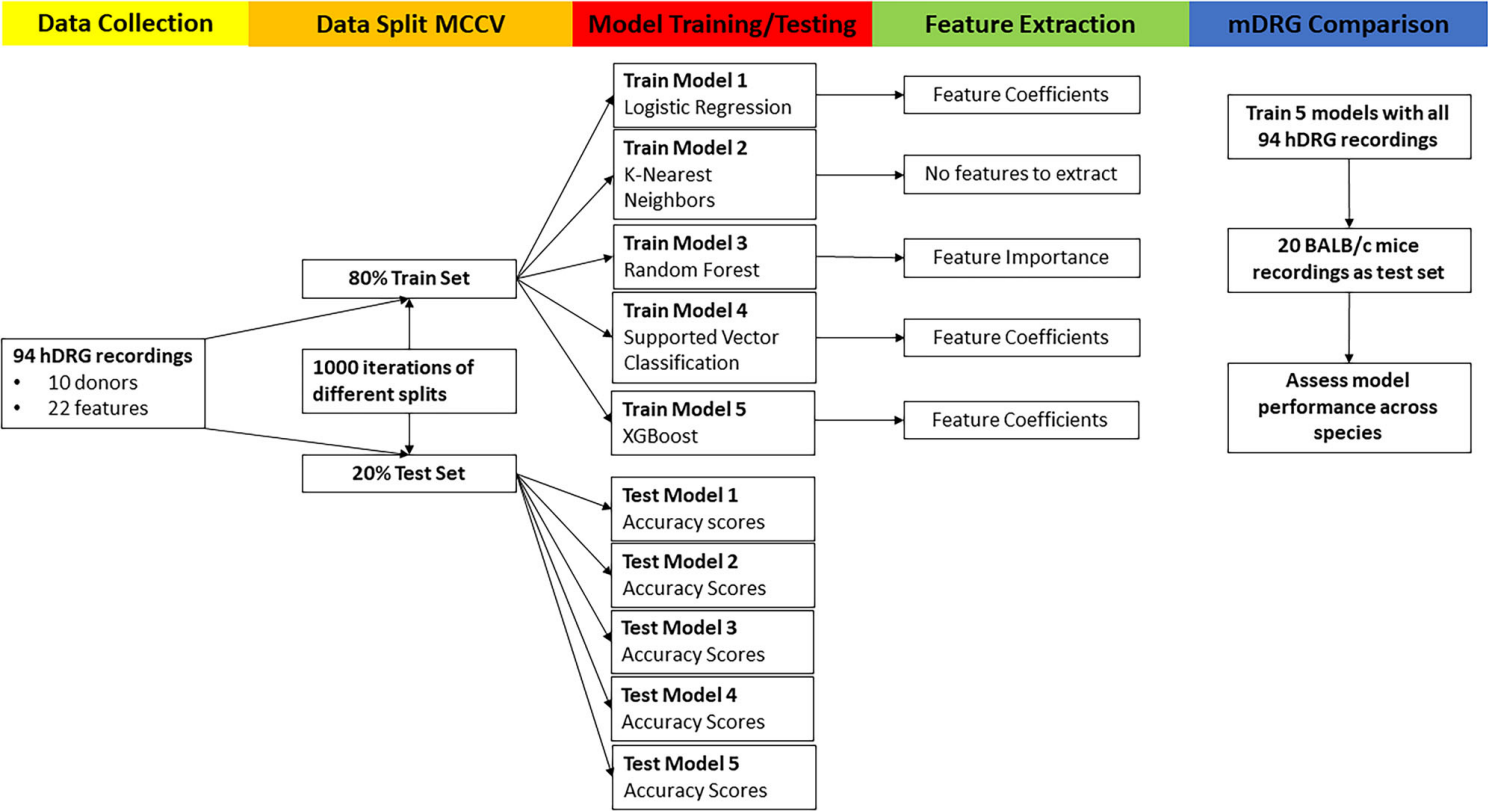
Machine learning model flow chart. Analysis was performed on 94 hDRG recordings, split using Monte Carlo cross-validation (MCCV), five different models run, and features extracted. Finally, we ran this analysis again using recordings of mouse DRG (mDRG).

**Figure 5.**
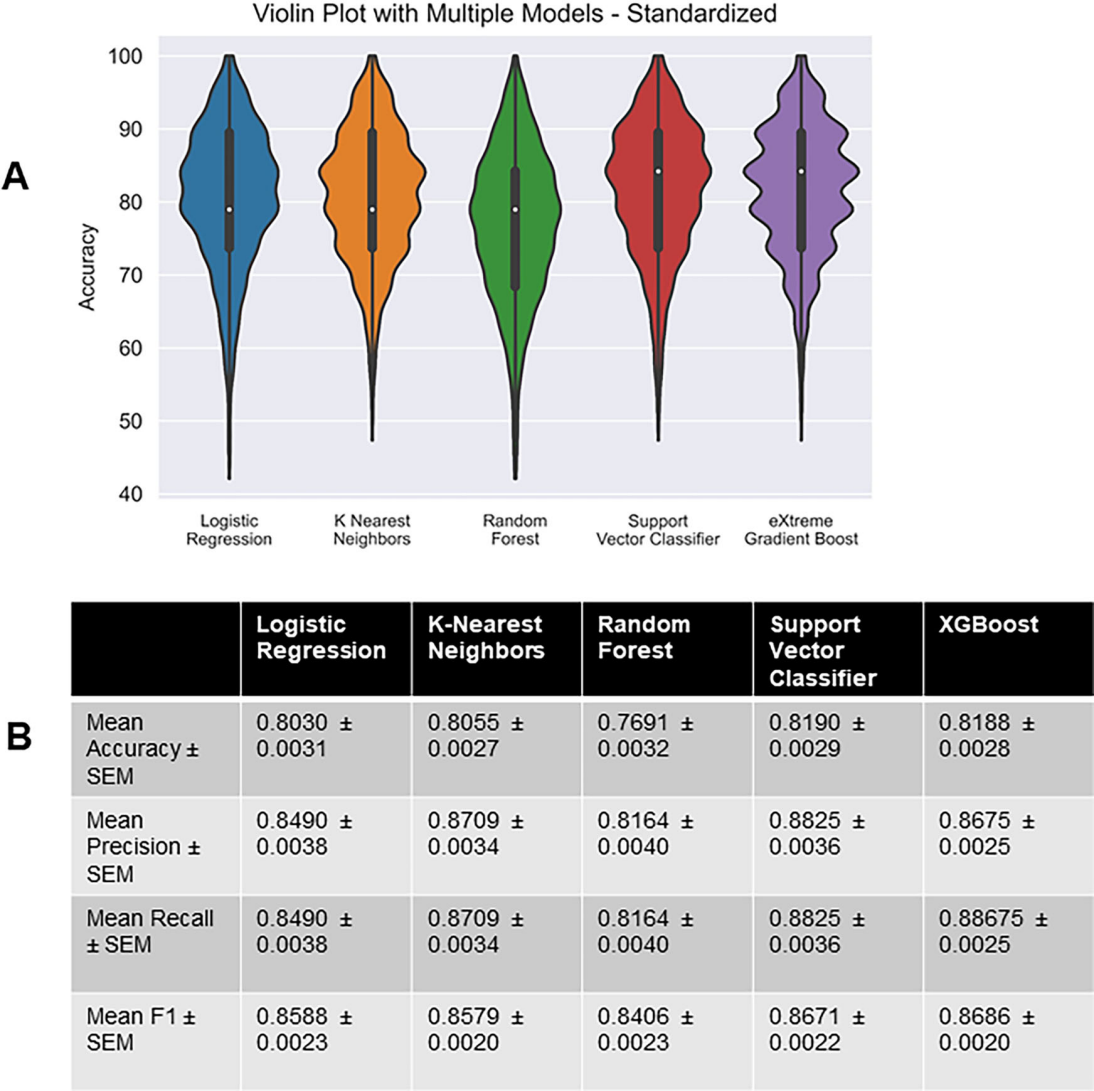
Performance of chosen models across randomized train–test splits. ***A***, Accuracies of logistic regression, *k*-nearest neighbors, random forest, linear support vector machine, and eXtreme Gradient Boost on Monte Carlo simulation (1,000 randomly created 80-20 train–test splits) were compared with violin plots of model accuracy when trained and tested on standardized hDRG-N data. Plot represents the range of accuracies, and interior box and whisker plot shows quartiles and median. ***B***, Table showing performance metrics of all models.

**Figure 6.**
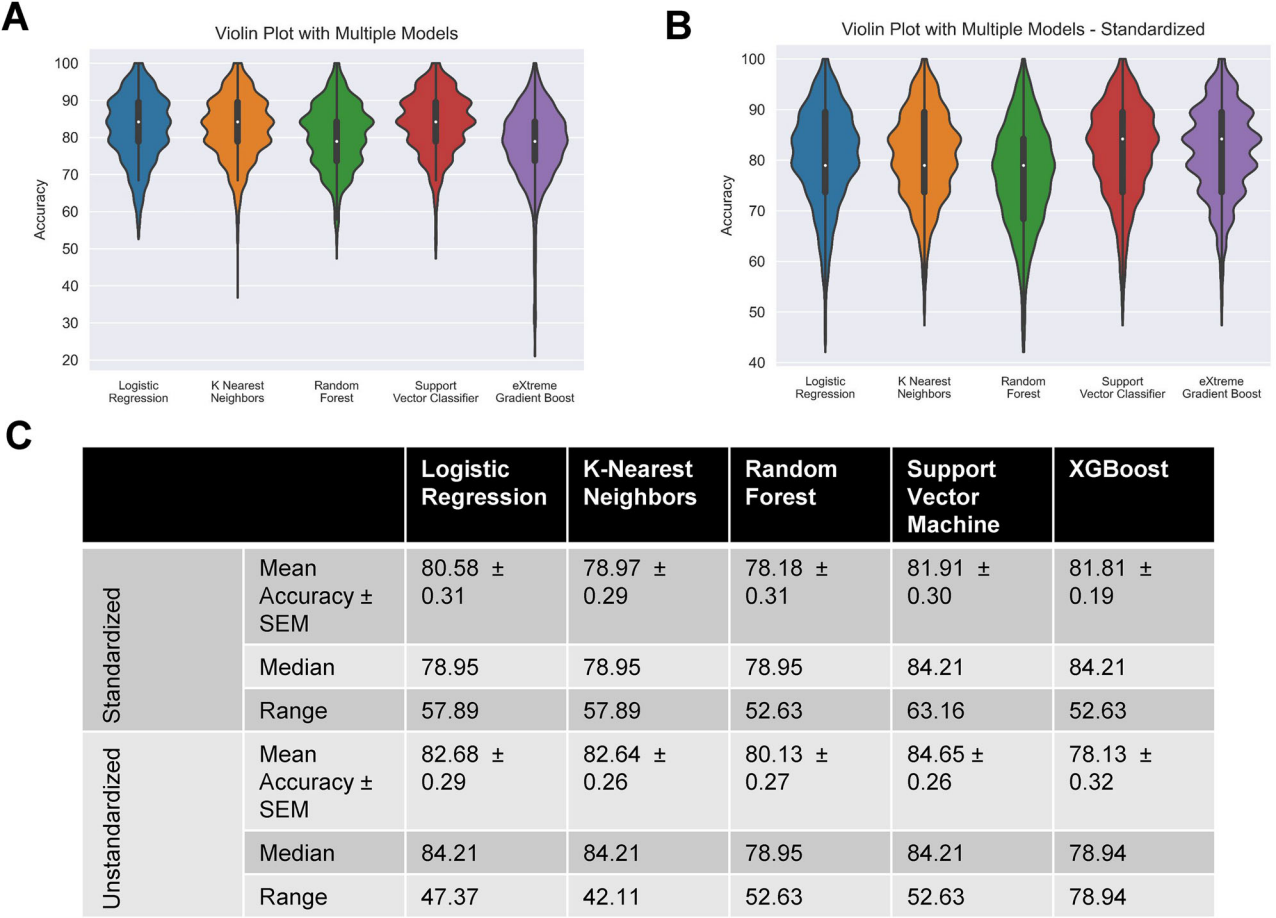
Performance of chosen models across randomized train–test splits. Performances of logistic regression, *k*-nearest neighbors, random forest, and linear support vector machine on Monte Carlo simulation (1,000 randomly created 80-20 train–test splits) were compared with violin plots of model accuracy when trained and tested on standardized/unstandardized dataset, hue represents model, plot is cut to represent the range of accuracies, and interior box and whisker plot show quartiles and median. ***A***, Unstandardized dataset. ***B***, Standardized dataset. ***C***, Table showing performance metrics of both groups of models.

**Figure 7.**
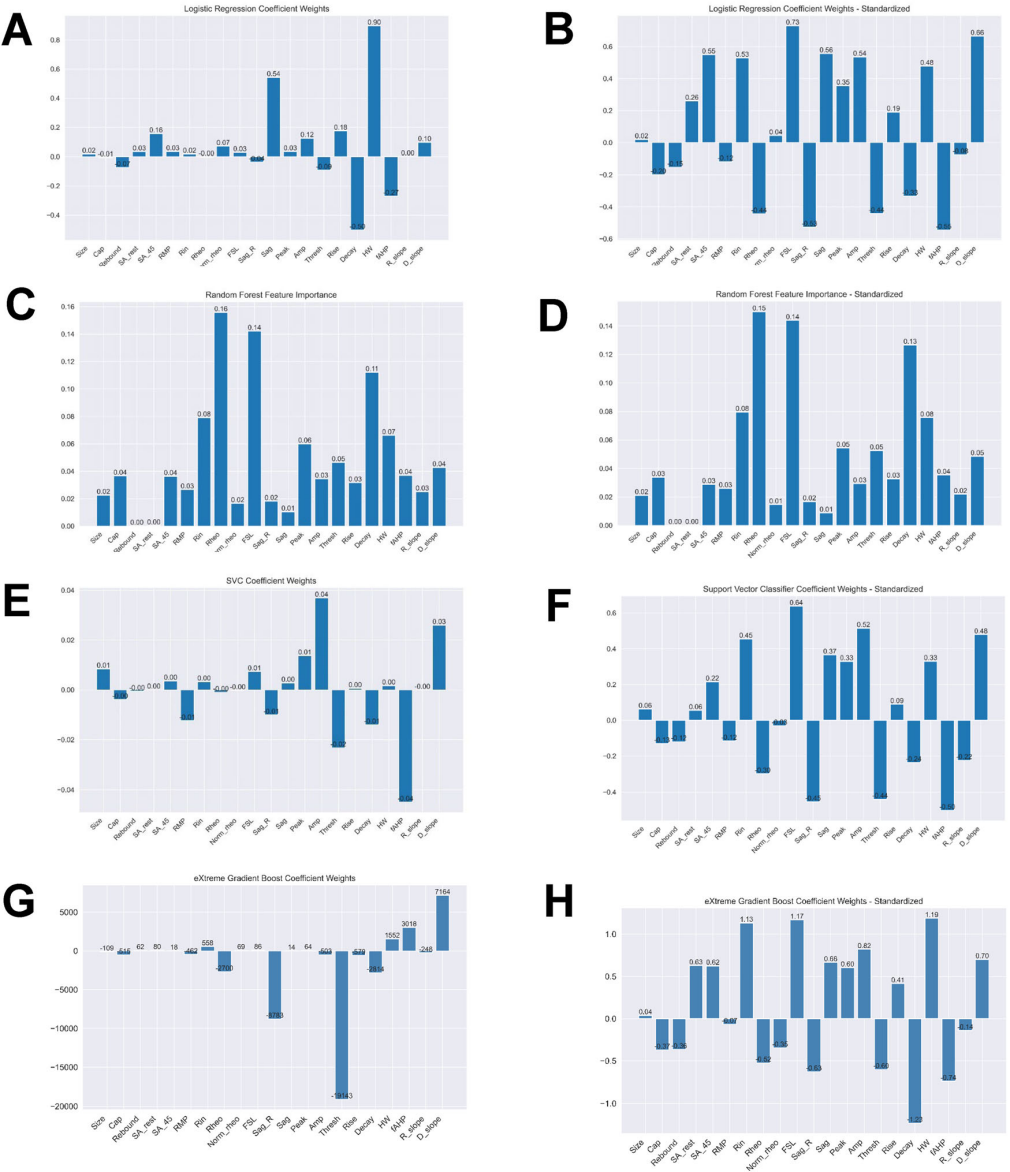
Feature importance metrics across logistic regression, random forest, and support vector classifier for standardized and unstandardized data. Mean coefficient weights or feature importance, when applicable, were extracted from the iterations in the Monte Carlo simulation. Graphs on the left represent models with unstandardized data while right use standardized. ***A***, ***B***, Logistic regression. ***C***, ***D***, Random forest. ***E***, ***F***, Support vector classifier.

To test if machine learning was able to predict multi- versus single-firing cells based on electrophysiological features, we recorded 94 hDRG-N from 10 donors. Since this is a relatively low number for machine learning models, we used a MCCV method that split the sample in an 80/20% train/test split across 1,000 iterations. Then we ran all the splits through five different models, generated model performance scores, and extracted feature coefficients and importances from each model (Fig. 4). We tested five machine learning models, logistic regression (LR), k-nearest neighbors (KNN), random forest classifier (RF), support vector classifier (SVC), and eXtreme Gradient Boost (XGBoost) with (Fig. 5) and without feature selection (Figs. 6, 7). Machine learning models such as LR and SVC have a hard time handling highly correlated features; therefore we ran the models with all features (Figs. 6, 7) and with feature selection that accounts for highly correlated variables (Fig. 5; Ranganathan et al., 2017). Several features that are highly correlated are the features describing AP duration and AP amplitude. Since both AP rise and AP decay time affect AP HW and all three were significantly longer in multi-firing cells (Fig. 2), we excluded AP rise time and AP decay time from the features used to determine model accuracy in our final models. Also, AP amplitude is highly correlated with AP threshold and AP peak. Since AP amplitude was not significantly different between single- and multi-firing cells but AP peak was, we decided to exclude AP amplitude and focus the models on AP peak. Rise slope and decay slope are direct measurements of the relationship of AP amplitude and AP duration, so those were also dropped from the final model predictions. Because our dataset is relatively small for machine learning algorithms, we ran 1,000 Monte Carlo simulations that randomized the 80/20 train/test split of the data. Model accuracies were plotted in a violin plot and model performance was compared (Fig. 5*A*,*B*). We found that all models had accuracies ∼80% on average (Fig. 5*B*). SVC and XGBoost were the best performing models with an accuracy of 81.90 ± 0.29% and 81.88 ± 0.28%, respectively. LR and KNN were the next best performing models with an accuracy of 80.30 ± 0.31% and 80.55 ± 0.31%. RF was the worst performing model with an accuracy of 76.91 ± 0.32%. RF is a popular model in biological predictive algorithms, but our data shows that multiple models should be tested to determine the best model for a dataset.

### Electrophysiological features that are predictive of single- and multi-firing hDRG-N

**Figure 8.**
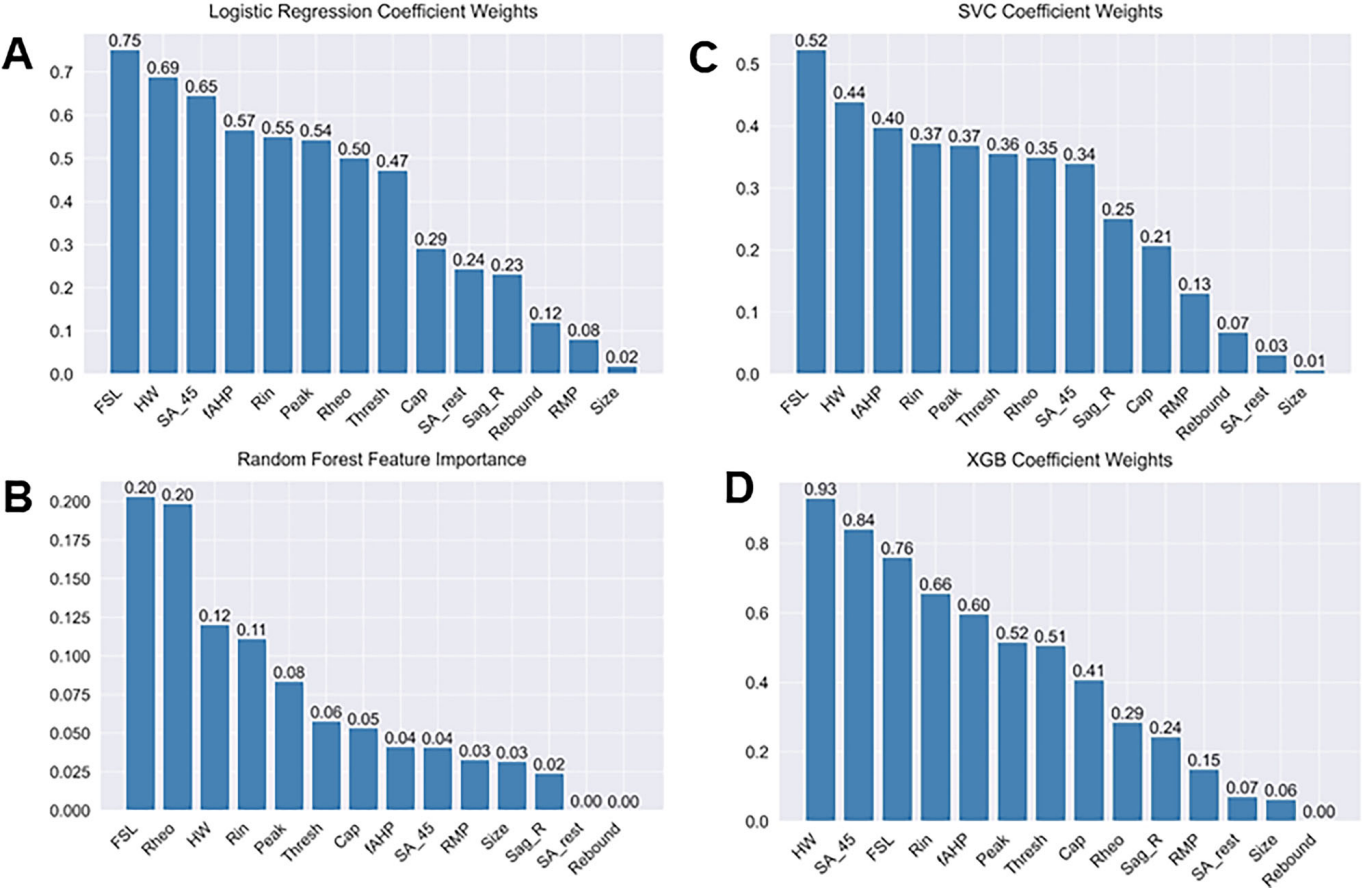
Feature importance metrics across logistic regression, random forest, and support vector classifier for standardized data. Mean coefficient weights or feature importance, when applicable, were extracted from the iterations in the Monte Carlo simulation and ordered. Absolute value of coefficients is displayed for logistic regression and support vector classifier. ***A***, Logistic regression. ***B***, Random forest. ***C***, Support vector classifier. ***D***, XGBoost.

**Figure 9.**
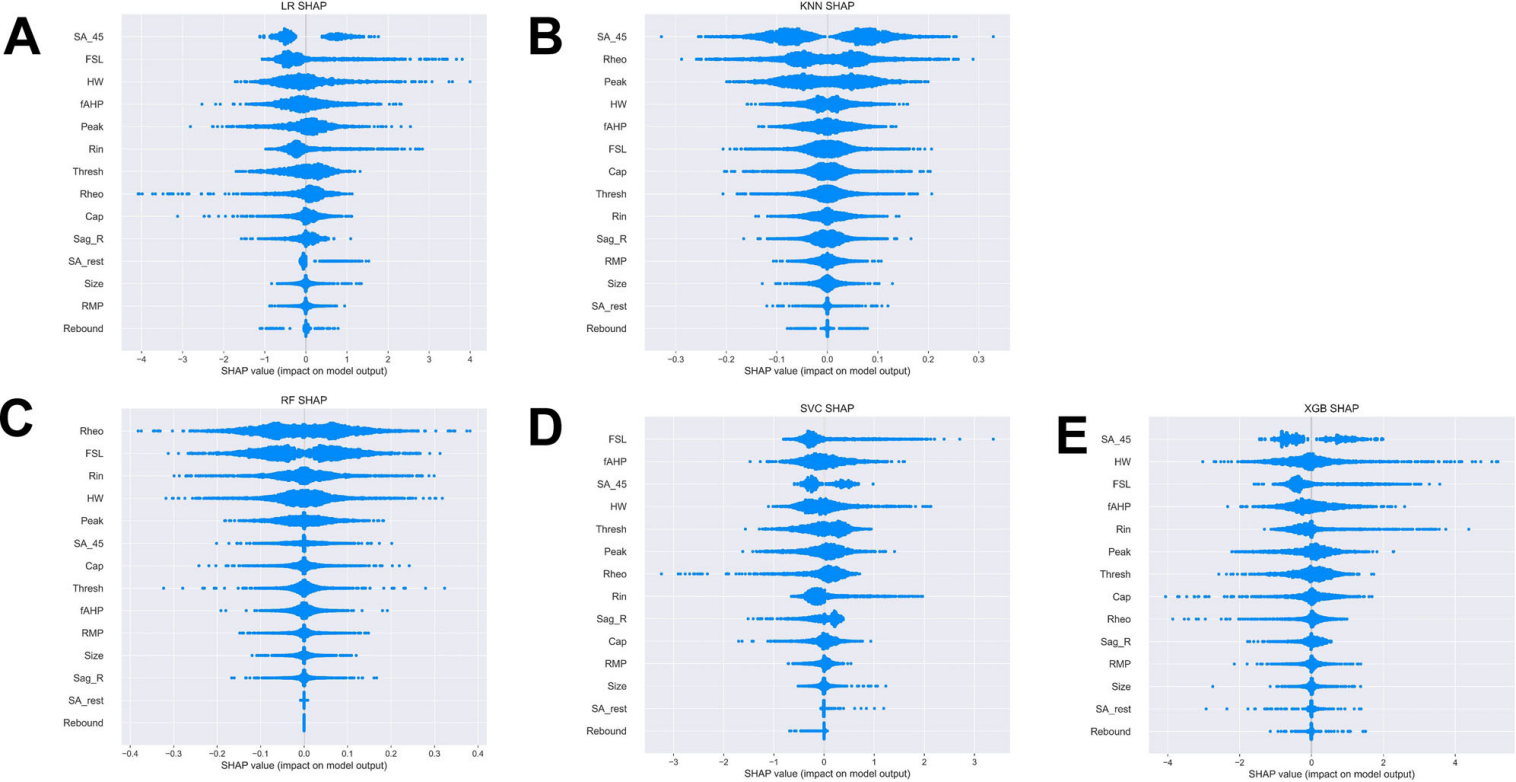
Monte Carlo Shapely Additive Explanations (SHAP) Beeswarm plots. Beeswarm plots for ***A*** logistic regression. ***B***, *k*-nearest neighbors. ***C***, Random forest. ***D***, Support vector classifier. ***E***, XGBoost.

**Figure 10.**
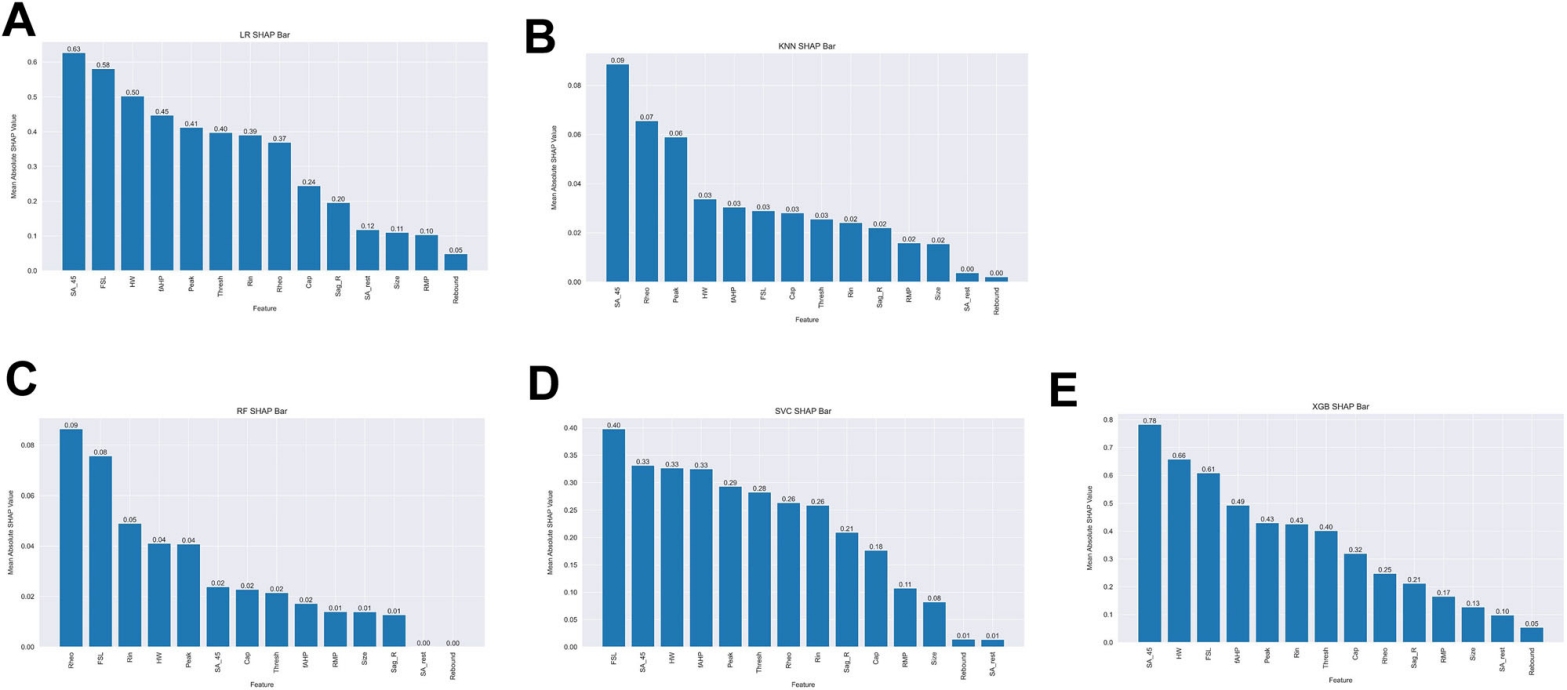
Monte Carlo Shapely Additive Explanations (SHAP) values. ***A***, Logistic regression. ***B***, *k*-nearest neighbors. ***C***, Random forest. ***D***, Support vector classifier. ***E***, XGBoost.

We next extracted feature coefficients for the LR and SVC models and feature importance for the RF model (Fig. 4). The KNN model is not able to compute to feature coefficients; therefore, it was not used to compute features predictive of multi-firing hDRG-N. Feature importance and coefficients are automatically calculated by the machine learning algorithms and can be extracted after model fitting. After running MCCV and generating our models, we extracted the resulting feature coefficients from the models to determine which features were most important in predicting single or multi-firing in hDRG-N. The LR, RF, and SVC model all predicted that a long FSL was the most important feature in predicting multi-firing cells while XGBoost predicted it as the third most important feature (Fig. 8). XGBoost predicted a long AP duration (measured by HW) was the most important feature while LR and SVC predicted it as the second most important feature for predicting multi-firing hDRG-N, while the RF model predicted it as the third most important feature. Other features that had relatively high coefficients/importance in predicting multi-firing included rheobase, SA_45, AHP, and *R*_in_. We also used Shapley Additive Explanations to extract feature importance for all the models, and while the relative importance of each feature changed slightly, the same features, a long FSL, a long HW, SA_45, and a low rheobase, all were scored highly in predicting multi-firing hDRG-N (Figs. 9, 10). Taken together, these data show that a long FSL and long HW are two of the most important features in predicting multi-firing hDRG-N.

### PCA extracts features important for multi-firing cells

**Figure 11.**
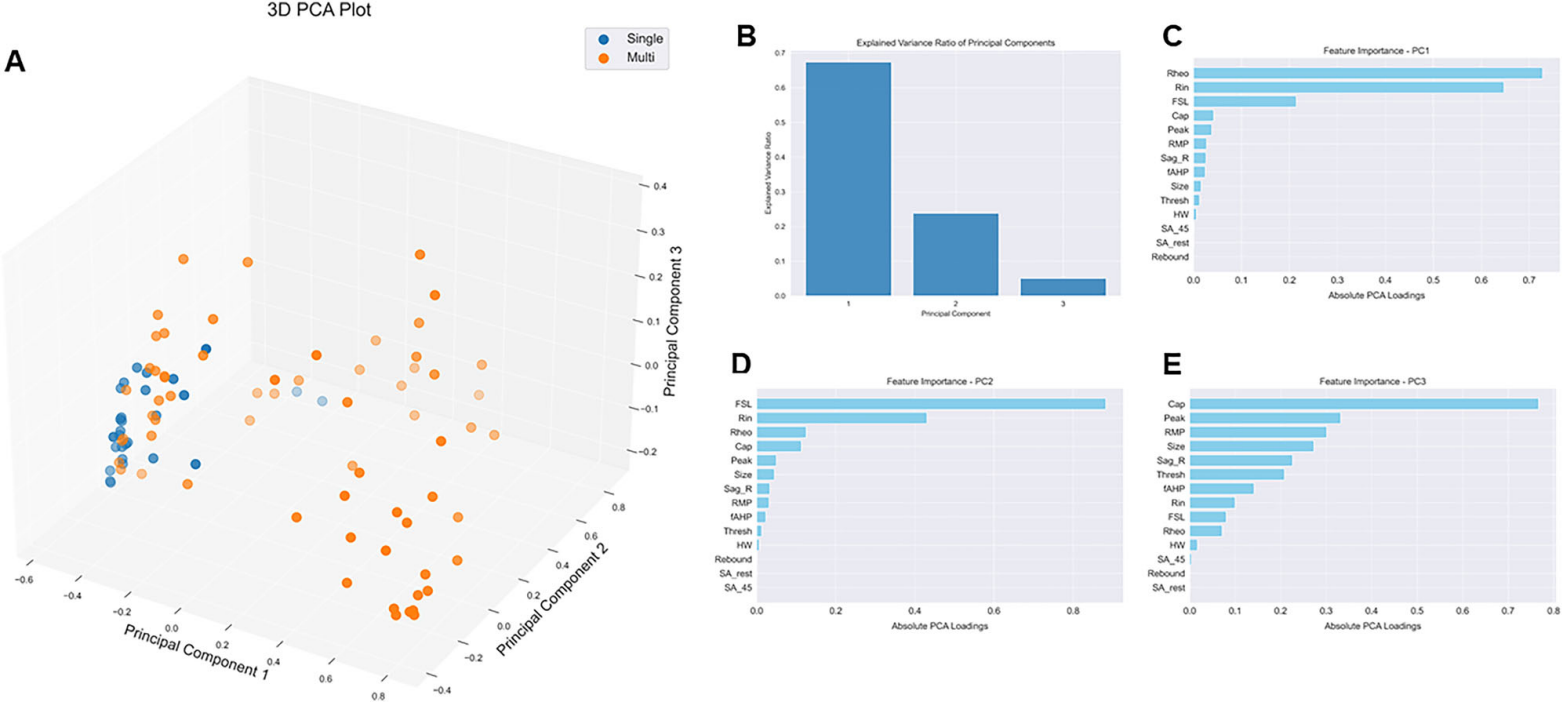
PCA with data normalization. ***A***, PCA plot. ***B***, Explained variance for each PC. ***C***, Feature importance for PC1. ***D***, Feature importance for PC2. ***E***, Feature importance for PC3.

We performed 3D PCA on our 94 hDRG-N using the features selected for the machine learning models. We found that there was a cluster of multi-firing cells that was distinct from the single-firing neurons (Fig. 11*A*, orange dots), but some multi-firing cells did cluster with the single-firing cells (Fig. 11*A*, blue dots). We found that 67.4% of the variance was explained by principal component 1 (PC1), 23.8% of the variance was explained by PC2, and that 5.1% of the variance was explained by PC3 (Fig. 11*B*). The feature importance contribution to each PC can be explained by the PC loadings. The loadings for PC1 were 0.73 for rheobase, 0.65 for *R*_in_, and 0.21 for FSL (Fig. 11*C*). The feature loadings for PC2 were 0.88 for FSL, 0.43 for *R*_in_, and 0.12 for Rheo (Fig. 11*D*). Since PC1 and PC2 explain 95% of the variance, the most important features for explaining the variance are rheobase, FSL, and *R*_in_.

### Machine learning models predictive of multi-firing in hDRG-N are able to predict multi-firing in mDRG-N

**Figure 12.**
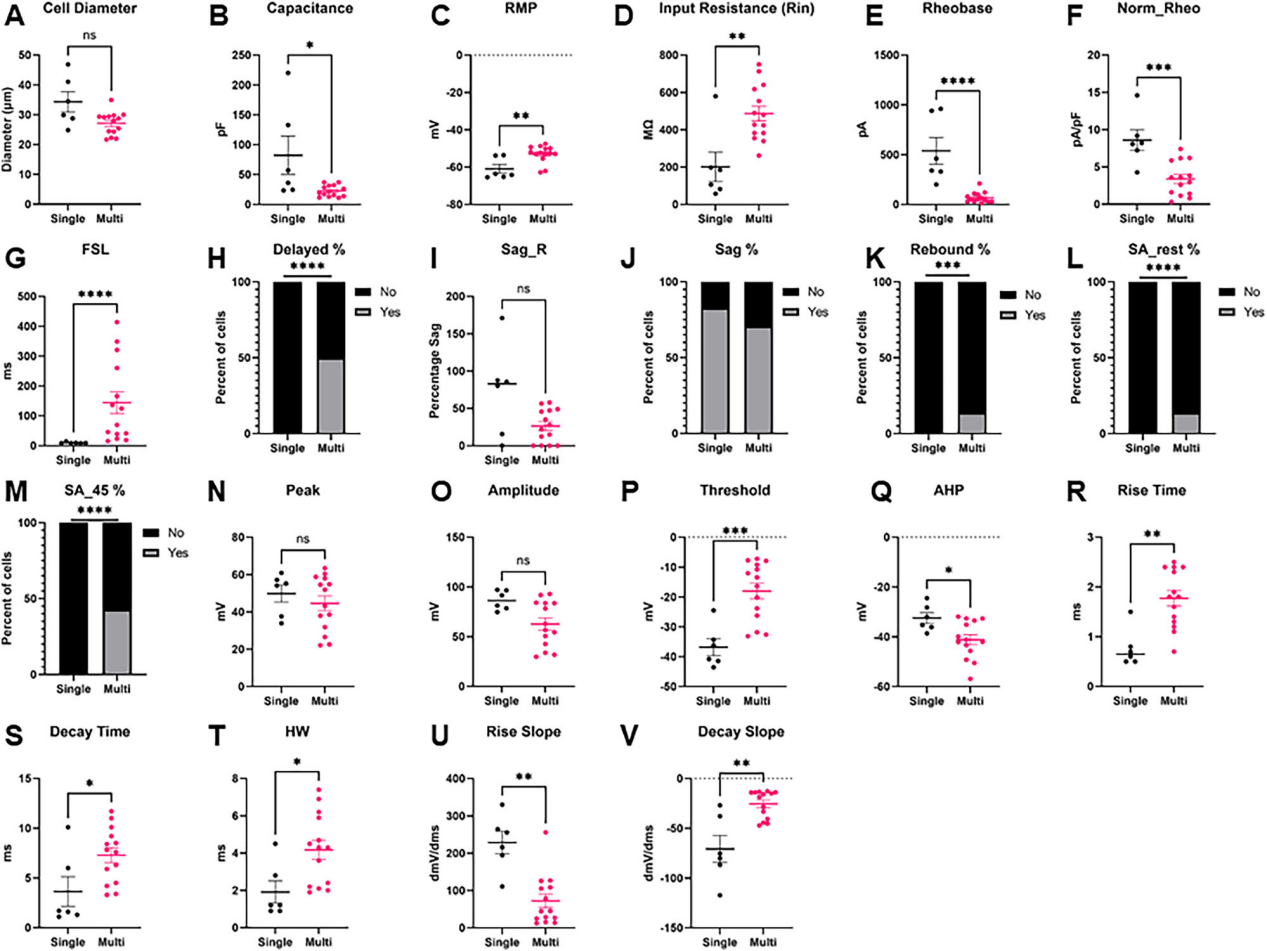
Intrinsic properties and firing phenotypes of mDRG-N neurons in single- versus multi-firing cells. Intrinsic properties and firing phenotypes were compared between single- and multi-firing cells. ***A***, Cell diameter, ***B***, capacitance, ***C***, resting membrane potential (RMP), ***D***, input resistance (Rin), ***E***, rheobase, ***F***, normalized rheobase, ***G***, first-spike latency (FSL), ***H***, percent delayed firing, FSL > 100 ms, ***I***, sag percentage at −100 pA current input (Sag_R), ***J***, percentage of cells with visible sag, ***K***, percentage of cells that have rebound firing. ***L***, Percentage of cells with spontaneous activity (SA) at rest, ***M***, percentage of cells with spontaneous activity when held at −45 mV. ***N***, AP peak. ***O***, AP amplitude. ***P***, AP threshold. ***Q***, AP after hyper-polarization (AHP). ***R***, AP rise time, ***S***, AP decay time, ***T***, AP half-width. ***U***, Max AP rise slope. ***V***, Max AP decay slope. *p < 0.05, **p < 0.01, ***p < 0.001, ****p < 0.0001 by Mann–Whitney for ***A–G***, ***I***, ***N–V***. By Fisher's exact test for ***H***, ***J–M***.

**Figure 13.**
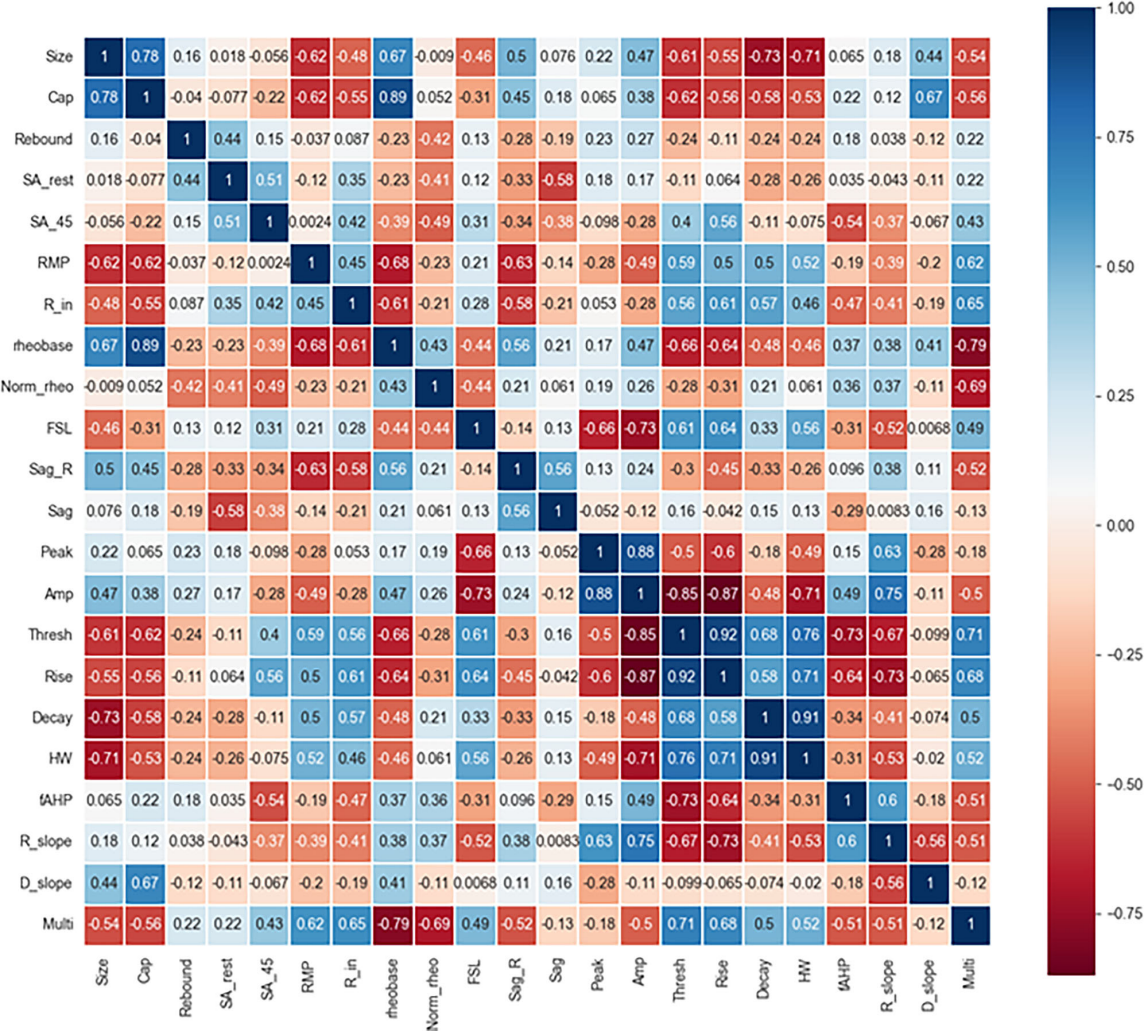
Pearson’s correlation for mDRG properties. Features compared included size, capacitance (Cap), presence of rebound firing, spontaneous activity at rest (SA_rest), spontaneous activity at −45 mV (SA_45), resting membrane potential (RMP), input resistance (Rin), rheobase (Rheo), normalized rheobase (Norm_rheo), first spike latency (FSL), sag ratio (sag_R), presence of visible sag, AP peak, AP amplitude, AP threshold, AP rise time, AP decay, AP half-width, afterhyperpolarization (fAHP), rise slope (R_slope), decay slope (D_slope), and multi-firing.

We found that similar to hDRG-N, ∼30% (6/20) of mDRG-N were single-firing in our dataset. To test if the models and features that are important in single- versus multi-firing hDRG-N are also important in mDRG-N, we first compared the electrophysiological feature differences in single- and multi-firing cells in mDRG-N (Fig. 12). We found several properties were significantly different in multi-firing mDRG-N such as a longer FSL, a lower rheobase, and a longer AP HW, similar to the properties that were different in hDRG-N. Pearson’s correlation matrix shows that these features were strongly correlated with multi-firing cells (Fig. 13). We found that the properties that were negatively correlated with multi-firing hDRG-N, such as rheobase, were also negatively correlated with mDRG-N and that properties positively correlated with multi-firing hDRG-N, such as FSL and HW were, also positively correlated in mDRG-N.

**Figure 14.**
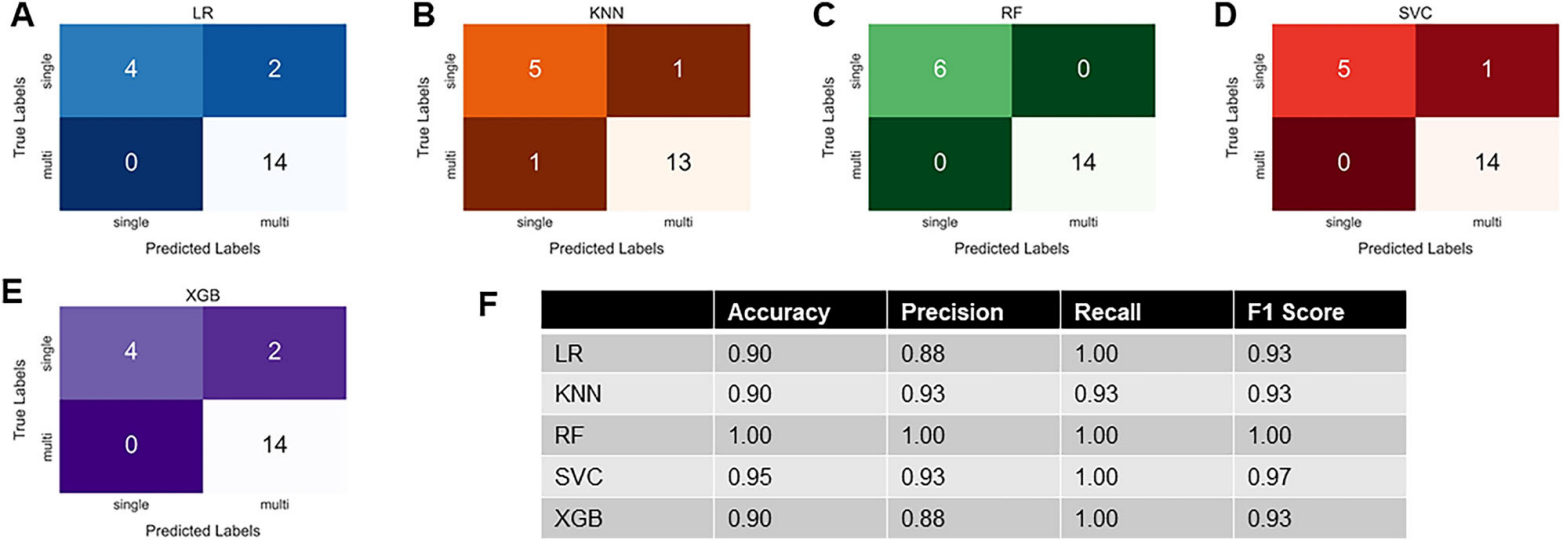
hDRG-N models are predictive of multi-firing in mDRG-N with high accuracy. Twenty mDRG-N recordings were applied to models developed for hDRG-N. We are showing the ***A*** confusion matrix for LR model. ***B***, Confusion matrix for KNN model. ***C***, Confusion matrix for the RF model. ***D***, Confusion matrix for SVC model. ***E***, Confusion matrix for XGBoost. ***F***, Accuracy, precision, recall, and *F*-1 scores for mDRG-N compared with each model.

Models generated using all 94 hDRG-N are able to predict single- and multi-firing in mDRG-N with >90% accuracy (Fig. 14*F*). We found that the RF hDRG-N generated model performed the best when applied to the mDRG-N dataset with 100% accuracy, precision, recall, and *F*1 scores (Fig. 14*C*, confusion matrix) while the SVC model performed second best with 95% accuracy, 93% precision, 100% recall, and 97% *F*1 score (Fig. 14*D*, confusion matrix). LR, KNN, and XGBoost all performed similarly with 90% accuracy (Fig. 14*A*,*B*,*F*). These data show that the same electrophysiological features that predict multi-firing in hDRG-N are predictive of multi-firing in mDRG-N as well.

## Discussion

Both hDRG-N and mDRG-N are used to understand the electrophysiological properties associated with pain. Multi-firing DRG-N are more excitable than single-firing DRG-N; therefore, we examined the electrophysiological properties that are predictive of multi-firing using machine learning. We found that a similar percentage of neurons were multi-firing in both hDRG-N and mDRG-N, ∼70%. We found that in both hDRG-N and mDRG-N, a long FSL and a long AP HW are the properties that are most predictive of multi-firing DRG-N. While many other features were significantly different in single- versus multi-firing cells, such as capacitance and AP peak, machine learning shows that these specific features were not the most predictive of multi-firing. Machine learning helps us identify which features are likely to be important in regulating multi-firing in a way that standard statistical methods cannot. We developed our machine learning models using our hDRG-N dataset which came from a diverse demographic of 10 donors. We found that several properties were negatively or positively correlated with multi-firing cells. For example, a long FSL, a long AP HW, and a low rheobase were correlated with multi-firing cells. We also used a smaller dataset of naive untreated BALB/c mice and found those same properties were correlated with multi-firing mDRG-N, though the correlations were much stronger. We hypothesize that since BALB/c share the same genetics and our sample looked only at naive mice killed the same way, the properties of mDRG-N are more consistent when compared with our diverse set of hDRG-N with a variety of genetics, disease states, cause of death, and in vivo treatments. While there may be some differences in the molecular mechanisms governing multi- and single-firing in hDRG-N and mDRG-N, our data shows that the same electrophysiological properties are predictive of multi-firing across both species. In fact, the machine learning models we generated for the hDRG-N had higher accuracy scores when the mDRG-N dataset was tested.

There are at least eight neuronal subtypes in the DRG that can be defined by genetic labeling, nonpeptidergic nociceptors, peptidergic nociceptors, proprioceptors, and five classes of low threshold mechanoreceptors (LTMR), each with distinct firing patterns (Zheng et al., 2019). Several of these are multi-firing, such as nociceptors, C-LTMR, and proprioceptors, while some are single-firing, such as Aβ SA1-LTMR and Aβ Field-LTMR. The nociceptors and C-LTMR have very similar firing patterns, long AP HW and a long FSL, while the Aβ SA1-LTMR and Aβ Field-LTMR have a short AP HW and short FSL. Peptidergic DRG neurons that have a higher capacitance have a smaller AP HW and are less likely to be multi-firing (Zheng et al., 2019). While we only looked at single-firing versus multi-firing neurons, future work could focus on using machine learning to detect different neuronal subtypes based on firing patterns.

It has been reported by others that multi-firing hDRG-N have a longer FSL and longer AP HW (Yi et al., 2022). Molecular experiments in neurons have attributed changes in AP HW to a variety of voltage-gated sodium channels (Na_V_), voltage-gated potassium channels (K_v_), and voltage-gated calcium channels (VGCC) and that these ion channels are important in neuronal excitability and pain perception (Alles and Smith, 2018; Goodwin and McMahon, 2021). Several Na_V_ channels have been implicated in pain in humans and animal models, including Na_V_1.7 and Na_V_1.8 (Alles and Smith, 2021; Goodwin and McMahon, 2021). Na_V_1.7 (HWTX-IV) and Na_V_1.8 (A-803467, PF-01247324) antagonists decrease neuronal excitability of sensory neurons (Payne et al., 2015; Ye et al., 2015; Atmaramani et al., 2020; Mulpuri et al., 2022), while activation of Na_V_1.8 increases neuronal excitability (Ye et al., 2015). Sensory neurons with increased Na_V_1.8 expression had longer AP HW (Djouhri et al., 2003; Thériault and Chahine, 2014; Zheng et al., 2019; Mulpuri et al., 2022). Knockdown of the β4 subunit of Na_V_1.8 decreased AP HW and decreased neuronal excitability in mDRG-N (Xiao et al., 2019). Single-cell RNA-seq has found that increased Na_V_1.8 and Na_V_1.9 expression and decreased Na_V_1.7 expression are correlated with an increase in AP HW (Thériault and Chahine, 2014). Expression of Na_V_1.8 and Na_V_1.9 is high in multi-firing nociceptors and C-LTMR and low in single-firing Aβ-LTMR (Zheng et al., 2019). Taken together these data suggest that Na_V_1.8 and possibly Na_V_1.9 increase AP HW and neuronal excitability, and that targeting Na_V_1.8 pharmacologically decreases AP HW and excitability. Our machine learning models show that increased AP HW is predictive of multi-firing cells and the literature suggests Na_V_1.8 may be a molecular mechanism that regulates both AP HW and neuronal excitability.

Several K_v_ channels are also implicated in regulating AP HW, FSL, and neuronal excitability. Blocking K_v_3 and K_v_1 channels with TEA and DTX increased the AP HW in hippocampal neurons (Hoppa et al., 2014). Experiments in rat DRG neurons show that dominant-negative mutation in K_v_3.4 effectively broadens AP HW showing that K_v_3.4 governs AP repolarization (Alexander et al., 2022). Rat DRG-N treated with calcineurin which attenuates (decreases) Kv3.4 action increases AP HW (Zemel et al., 2017). Data shows that multi-firing nociceptors and C-LTMR have a long AP HW and a long FSL (Zheng et al., 2019). These neuronal subtypes showed decreased levels of K_v_1.1, K_v_1.2, K_v_2.1, K_v_3.1, K_v_3.3, K_v_7.2, K_v_7.3, K_v_9.1, and K_v_11.1 and increased levels of K_v_4.1, K_v_6.2, and K_v_6.3 when compared with single firing Aβ-LTMR (Zheng et al., 2019). Using a variety of K_v_ inhibitors, the main contribution to potassium currents in nonpeptidergic nociceptors were K_v_1, K_v_2, and K_v_4, while in C-LTMR the main contributor was K_v_4 (Zheng et al., 2019). In single-firing Aβ-LTMR the main contributor to potassium currents was K_v_3 followed by K_v_1 (Zheng et al., 2019). Computational modeling showed that C-LTMR neurons without Kv4 would have a short FSL. Blocking K_v_4.3 with AmmTx3 or K_v_4.3 KO mice had a decreased FSL in C-LTMR, while blocking K_v_1 with DTx had no effect on firing properties in C-LTMR (Zheng et al., 2019). Blocking K_v_1 current with DTx in single-firing Aβ-LTMR turned them into multi-firing neurons (Zheng et al., 2019).

Calcium channels may also be involved in regulating FSL, AP HW, and neuronal excitability. The voltage-gated calcium channel alpha-2-delta-1 subunit (α_2_δ-1) has been implicated in chronic pain and neuronal excitability (Newton et al., 2001; Alles and Smith, 2018; Dolphin, 2018; Cui et al., 2021). Experiments in mDRG-N show that DRG cultured from α_2_δ-1 knock out mice have a shorter FSL and AP HW and that these neurons are less excitable, i.e., firing fewer APs (Margas et al., 2016). Ca_V_2.2 and α_2_δ-1 expression is also increased in multi-firing nociceptors and C-LTMR with long AP HW and long FSL, while it is absent in single Aβ-LTMR with short AP HW and short FSL (Zheng et al., 2019). Gabapentinoids are used as first-line treatment for neuropathic pain and act on α_2_δ-1 (Alles and Smith, 2018). Many publications show treatment with gabapentinoids decrease evoked calcium currents and neuronal excitability (Bannister et al., 2011; Biggs et al., 2014, 2015). The calcitonin gene-related peptide (CGRP) was also increased in multi-firing nociceptors and C-LTMR (Zheng et al., 2019).

Several other ion channels are implicated in nociception and chronic pain including HCN2, T-type Ca^2+^ channels, and Ca^2+^-sensitive K^+^ channels (Emery et al., 2012; Alles and Smith, 2021). Importantly, HCN2 channels modulate sag ratio and rebound firing, which we found were correlated with multi-firing cells by Pearson’s correlation analysis (Emery et al., 2011). Increased expression of T-type Ca^2+^ channel Ca_V_3.2 is associated with increased spontaneous activity which we also found was present almost exclusively in multi-firing cells (Li et al., 2017). Many other channels are implicated in neuronal excitability and warrant future studies in how these affect electrophysiological properties.

There are extensive data demonstrating that a variety of ion channels affect AP HW and FSL. Our machine learning suggests that these are the most predictive features of single- versus multi-firing in DRG-N. Much of the work to elucidate which ion channels are important for these features has been done in rodents and not hDRG-N. Given the machine learning models for hDRG-N are also able to predict multi-firing in mDRG-N and that the same electrophysiological features are correlated with multi-firing cells in both species, we predict the same ion channels would be important in hDRG-N. To test this hypothesis, single-cell patch-RNA-seq and/or post hoc labeling could be used to determine which ion channels are up- or downregulated in multi-firing hDRG-N. Additionally, it has been shown that there are at least eight neuronal subtypes in DRG-N, with a variety of firing patterns that can be differentiated by genetic markers (Zheng et al., 2019; Qi et al., 2024). Our current dataset and machine learning tools cannot differentiate these neuronal subtypes presently. However, we hope to continue expanding our capabilities in future studies.

## Synthesis

Reviewing Editor: Masha Prager-Khoutorsky, McGill University Decisions are customarily a result of the Reviewing Editor and the peer reviewers coming together and discussing their recommendations until a consensus is reached. When revisions are invited, a fact-based synthesis statement explaining their decision and outlining what is needed to prepare a revision will be listed below. The following reviewer(s) agreed to reveal their identity: Christoforos Tsantoulas, Michael Hildebrand. Note: If this manuscript was transferred from JNeurosci and a decision was made to accept the manuscript without peer review, a brief statement to this effect will instead be what is listed below.

Synthesis of Reviews:

Both reviewers acknowledged that the findings are very interesting and provide important advances in our understanding on nociceptive neurons.

However, there are several issues that must be addressed before the manuscript can be published.

1. The translation implications of these findings, specifically the potential "to elucidate better targets for treatments of chronic pain" seem unclear. The authors should include a discussion addressing this issue and/or modify their statements with regards to implications into drug discovery.

2. Additional methodological details, example traces, and analyses should be included, as detailed in reviewers' comments.

3. Applying the method to a sensitised mouse DRG system to enhance translatability could significantly improve the manuscript. However, since this would require experiments using a different model system, this suggestion remains optional and was not deemed essential for inclusion in the manuscript."

Please refer to more detailed criticisms outlined below:

---------------------------------------------

Reviewer#1:

1) The idea is interesting, the experiments and analysis are carried out carefully and the manuscript is well written. My main criticism is that despite the statement that results "could elucidate better targets for treatment of chronic pain", there is a discontinue with regards to clinical application. While the use of human samples is a significant strength, it is not clear how the results may yield new molecular targets/approaches for treatment. The thorough discussion successfully links measured parameters with underlying ion channels counterparts, however these targets have already been extensively investigated as analgesic treatments. Thus, while the associations are of great biological/educational interest, the possibility of triggering novel clinical development is limited. Instead, perhaps the most immediate contribution to drug discovery would be to inform compound characterisation as part of early-stage in vitro development.

2) Another concern is that all experimental data are derived from sensory neurons in a physiological (acute) setting. We have no way of knowing whether these uncovered predictions would also apply to a 'sensitised' neuron from a chronic pain patient/model; in these states silent neurons can become spontaneously active, and single-firing neurons may produce trains of spikes. It would be particularly interesting to address this by performing some additional experiments in a sensitised system such as mouse DRGs derived from a pain model or cultures treated with inflammatory mediators. Such work would greatly enhance translatability and impact of the work.

3) A further improvement would be to perform the analysis in sub-populations of sensory neurons (e.g. small C-fiber vs large A-fibers) as this could enhance power to detect nociception-specific attributes.

4) Finally, the authors have done a good job reviewing the ion channel landscape but have omitted HCN2 channels which have been shown to not only affect AHP but also shape sensory neuron excitability in a variety of pain states.

Reviewer#2:

In this study by Zurek and colleagues, the researchers extensively characterize the passive membrane, excitability, and action potential waveform properties of human DRG neurons. The cell populations are divided between single firing and multi firing DRG neurons, with direct statistical comparisons for all properties between these two groups. The authors then use a combination of machine-learning and Principal Component Analysis approaches to test which properties are predictive of multi-firing, which are identified as a long decay time, low rheobase, and long first spike latency. This study is well executed, with high quality datasets that support the conclusions of the paper. Given the highly translational nature of these recordings on human nociceptors, this paper will be of interest to a broad range of basic science and pharmaceutical researchers. I have the below minor comments to further strengthen this study.

Comments:

1. The Methods should include key experimental details rather than citing past papers, such as the intracellular pipette solution composition and whether the authors calculated and corrected for liquid junction potentials.

1b. Whether LJP was corrected for or not is important, as it will impact the stated holding membrane potentials that are included in the results. Ideally, the LJP would be calculated based on in the internal and external solution compositions and then corrected for, which can be done post-recording.

1c. For extensive IV protocols with depolarizing steps up to 4000 pA, the authors should state the time and resting membrane potential between each sweep, as this will impact how much the voltage-gated channels can recover from inactivation between these highly depolarized steps.

2. Showing representative traces at a few example hyperpolarizing and depolarizing test potentials for single- versus multi- firing hDRG neurons at the beginning of Figure 1 would help illustrate the differences in firing patterns, rheobase, input resistance, and other biophysical properties between these distinct populations of cells (ie. - example source raw data for parameters in Figures 1A-M).

2b. The first action potentials for example spikes from each population could then also be zoomed in on to illustrate AP-related properties such as rise and decay slopes and AHP (ie.- example source raw data for parameters in Figure 1N-V). This would help the readers grasp how these values were obtained and what they could mean in terms of their impact on excitability.
